# miR2Diabetes: A Literature-Curated Database of microRNA Expression Patterns, in Diabetic Microvascular Complications

**DOI:** 10.3390/genes10100784

**Published:** 2019-10-09

**Authors:** Sungjin Park, SeongRyeol Moon, Kiyoung Lee, Ie Byung Park, Dae Ho Lee, Seungyoon Nam

**Affiliations:** 1 College of Medicine, Gachon University, Incheon 21565, Korea; oscarpark@gachon.ac.kr; 2 Gachon Institute of Genome Medicine and Science, Gachon University Gil Medical Center, Incheon 21565, Korea; 3 Department of Health Sciences and Technology, Gachon Advanced Institute for Health Sciences and Technology, Gachon University, Incheon 21565, Korea; moonsr1982@gmail.com; 4 Department of Internal Medicine, Gachon University Gil Medical Center, Incheon 21565, Korea; kylee@gilhospital.com (K.L.); park712@gilhospital.com (I.B.P.); 5 Department of Internal Medicine, Gachon University School of Medicine, Incheon 21565, Korea; 6 Department of Life Sciences, Gachon University, Seongnam 13120, Korea

**Keywords:** miRNA, diabetes mellitus, diabetic microvascular complications, databases

## Abstract

microRNAs (miRNAs) have been established as critical regulators of the pathogenesis of diabetes mellitus (DM), and diabetes microvascular complications (DMCs). However, manually curated databases for miRNAs, and DM (including DMCs) association studies, have yet to be established. Here, we constructed a user-friendly database, “miR2Diabetes,” equipped with a graphical web interface for simple browsing or searching manually curated annotations. The annotations in our database cover 14 DM and DMC phenotypes, involving 156 miRNAs, by browsing diverse sample origins (e.g., blood, kidney, liver, and other tissues). Additionally, we provide miRNA annotations for disease-model organisms (including rats and mice), of DM and DMCs, for the purpose of improving knowledge of the biological complexity of these pathologies. We assert that our database will be a comprehensive resource for miRNA biomarker studies, as well as for prioritizing miRNAs for functional validation, in DM and DMCs, with likely extension to other diseases.

## 1. Introduction

Diabetes mellitus (DM) is a critical societal and practical burden, both in developed and developing countries. In particular, DM may advance into various diabetic microvascular complications (DMCs), depending on disease duration, blood glucose impairment, diet, inactivity, and genetic background [1]. In 2015, the International Diabetes Federation predicted that 415 million individuals aged 20–79 years, experience DM, with a direct, worldwide economic burden of $673 billion USD [2]. By 2040, DM prevalence is predicted to continuously increase, affecting 642 million people [2,3]. Even direr, DM mortality, in 2015, amounted to 5.0 million deaths per year [3]. These substantial upsurges in DM incidence (even now, afflicting 1 in 11 persons) are believed to largely result from increasing rates of obesity and lack of physical activity [1]. 

Recently, microRNAs (miRNAs) have been found to play important roles in DM and DMC outcomes, thus representing promising prognostic biomarker candidates, as well as revealing possible mechanisms of pathogenesis of DM and DMCs [4,5,6]. In fact, several blood-borne miRNAs, including miR-15a, miR-29b, -126, and -28-3p significantly associate with the progression of type 2 diabetes mellitus (T2DM) [7]. Other blood-borne miRNAs, miR-21a and miR-93, associate with the advancement of type 1 diabetes mellitus (T1DM) [8]. In diabetes, and its complications, miRNAs are suggested to have various functions in hyperglycemia, pancreatic β-cell destruction, fibrosis, endothelial cell damage, and inflammation [9]. Moreover, urinary miRNAs are attractive noninvasive prognostic biomarkers of chronic kidney diseases, including diabetic nephropathy [10,11].

To collect such information, the bioinformatics community has supported a few manually curated miRNAs-disease relationship databases. For example, miR2Disease [12] is one of the earliest miRNA-disease information sources, based on manual curation. That database is limited by having only 15 entries available for DM and DMCs. In databases, such as OncomiRDB [13] and miRCancer [14], manual biocuration of cancer-related miRNAs have been fairly well accepted. The miRegulome [15] provides integrative miRNA regulatory modules for pathophysiology in diseases. But, an integrated resource on the miRNA regulome in DM is currently not available. Following cancer and cardiovascular disease, DM is the third-most prevalent disease in the world, even while extensively biocurated DM-related miRNA databases have yet to be constructed.

In this study, through systematic literature inspection of miRNAs, in DM and DMCs, we established miR2Diabetes, a new and up-to-date database for manually curated, DM-associated miRNAs. This database contains 325 manually biocurated miRNA-based experimental outcomes, for 156 miRNAs, and spans 14 distinct DMCs, including diabetic neuropathy, diabetic nephropathy, diabetic retinopathy, microalbuminuria, and others. In addition, the database supports not only multiple species (including model organisms) but also diverse tissue and fluid samples, including plasma, liver, pancreatic islets, and kidney. We believe that this database will be a valuable asset for both the study of miRNAs as biomarkers, and laboratory-based studies of miRNA functionality in the disease states of DM and DMCs.

## 2. Materials and Methods

### 2.1. Data Collection and Database Content

We gathered biomedical literature for data entry into our database, miR2Diabetes, from public databases, through July 31, 2019. Literature sources for the database were PubMed, Web of Science, and the Cochrane Library. Searched keywords were combinations of ‘diabetes’, ‘miRNA’, ‘microRNA’, ‘microvascular’, ‘nephropathy’, ‘neuropathy’, and ‘retinopathy’, resulting in 387 publications. After removing duplicates, we retrieved and assessed full-text articles. We then selected specific literature that quantified miRNA expression levels as related to DM and DMCs (including diabetic nephropathy, diabetic retinopathy, and diabetic neuropathy). We excluded articles that merely inspected miRNA single nucleotide variations (SNVs), and studies of controlled miRNA expression (e.g., knockdown or knockout). We collected miRNA dysregulation patterns, associated with DM and DMCs, with p-values of less than 0.05. We further extracted details from articles’ experimental designs, and their subsequent results, as follows: miRNA symbols, aliases, annotations, and reference metadata (e.g., PubMed ID, author, title), expression values or odds ratios (ORs), experimental subjects, related DMC phenotypes, and measurement types (e.g., qRT-PCR, microarray).

### 2.2. Nomenclature Standardization

We next standardized miRNA symbols, following the HUGO Gene Nomenclature Committee (HGNC) [16], from miRNA names, as declared in the original articles. When authors referred to mature miRNAs (e.g., hsa-miR-126-3p), we entered all miRNA symbols by their HGNC gene symbols (e.g., *MIR126*), into our database. Then, we changed that specific miRNA to its precursor miRNA form (e.g., hsa-*miR-126*) as its alias. We also gathered accession identifiers, from the HGNC database [17], for human gene names and used miRBase [18] to provide compatibility with other miRNA-related resources. 

We also standardized sample names and disease names in the literature collection, by using the BRaunschweig ENzyme DAtabase (BRENDA) Tissue and Enzyme Source Ontology [19], and The Disease Ontology [20], respectively. From these, we converted the samples and disease names to their hierarchically structured, controlled, ontology terms. In addition, we provide miRDB [21] links for miRNA functional targets. 

### 2.3. miRNA Dysregulation Pattern Generalization

We generalized terms of the collected miRNA dysregulation pattern as ‘UP’ or ‘DOWN’ when authors reported experimental outcomes as fold-changes (FCs) or odds ratios (ORs). When the FC or OR was greater than 1.3, compared to the case group (e.g., DMC patients) over control group (e.g., healthy controls), we categorized them as ‘UP’. If those values were less than 1/1.3, we considered these as ‘DOWN’. Experimental outcomes that showed up- and/or down-regulated miRNA expression were curated accordingly.

### 2.4. Database and Web Application Implementation

We constructed miR2Diabetes on Oracle™ MySQL 5.5 database management system, with utf8 as its default character set. All database data definition languages, and data manipulation languages, were written in ANSI-SQL. We also visualized miR2Diabetes contents, in our web application, using JSP™ 2.3/Servlet™ 3.1. The miR2Diabetes database is freely available [22], with no login requirement.

## 3. Results

### 3.1. Database Content and Statistics

Our miR2Diabetes database is a comprehensive repository for DM- and DMC-dysregulated miRNAs, providing information such as HGNC miRNA official symbols [17], miRbase [18] miRNA accessions, aliases, annotations, experimental designs, experiment results, dysregulation patterns, complication names, experimental subjects, and publication references.

**Figure 1:**
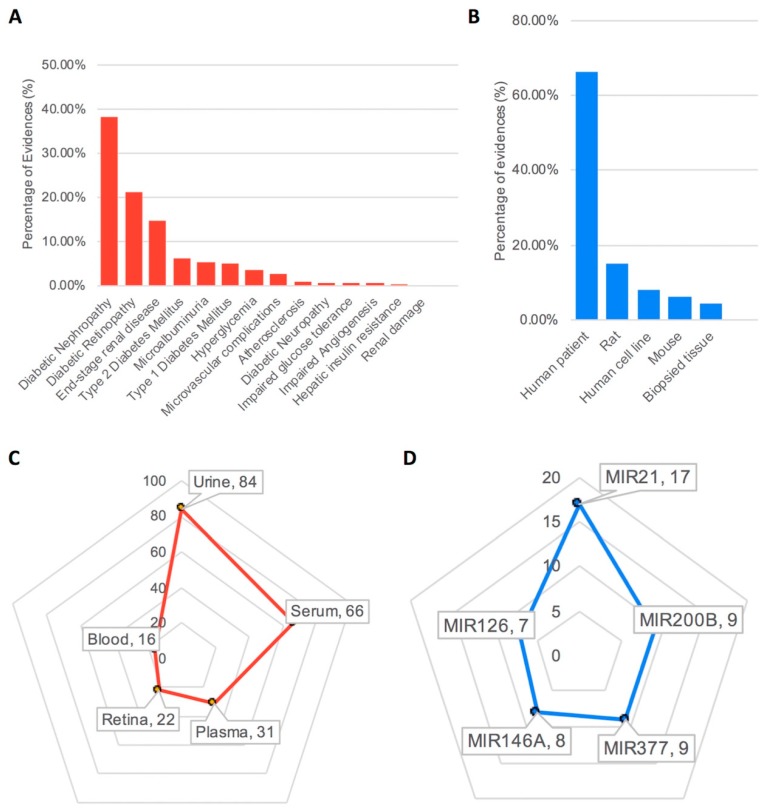
miR2Diabetes statistics. (**A**) Percentages of publication evidence contained in miR2Diabetes related to certain diabetic microvascular complications. (**B**) Percentages of miR2Diabetes publications related to specific organisms/tissues. (**C**) A pie chart of the top 5 experimental samples connected to miRNA dysregulation observations. (**D**) A pie chart of the top 5 miRNAs contained in our database.

In total, we screened 387 articles, resulting from specific and narrow search keywords, extracting 14 DMC types and 27 experimental sample types (including patient samples). Moreover, we deposited 325 miRNA dysregulation patterns (experimental outcomes), with 156 microRNAs, as related to various experimental designs. Among 325 experimental outcomes, “Diabetic Nephropathy”-related entries amounted to 38.15%, “Diabetic Retinopathy,” 21.23%, “End-stage renal disease,” 14.77%, followed by “Type 2 Diabetes Mellitus” and nine other DMCs (Figure 1A). Of 66.46% miRNA dysregulation patterns, measured from human patient samples, rat and human cell line experiments were 15.08% and 8.0%, respectively. MicroRNA expression levels, in mouse and human biopsied tissue, were only 6.16% and 4.31%, respectively (Figure 1B). The top five experimental sample sources used in measuring miRNA dysregulation are described in Figure 1C. The most prevalently assessed sample sources were urine (84 entries) and serum (66 entries). The three remaining sample sources were from similar numbers of observations (31 plasma, 22 retina, and 16 pancreatic islet samples, as labeled in our experimental outcome table). The most observed dysregulated miRNA was miR-21 (17 experimental outcomes) followed by miR-200B, miR-377, miR-146A, and miR-126, comprising the top five miRNAs in our database (Figure 1D).

### 3.2. Database Model

**Figure 2:**
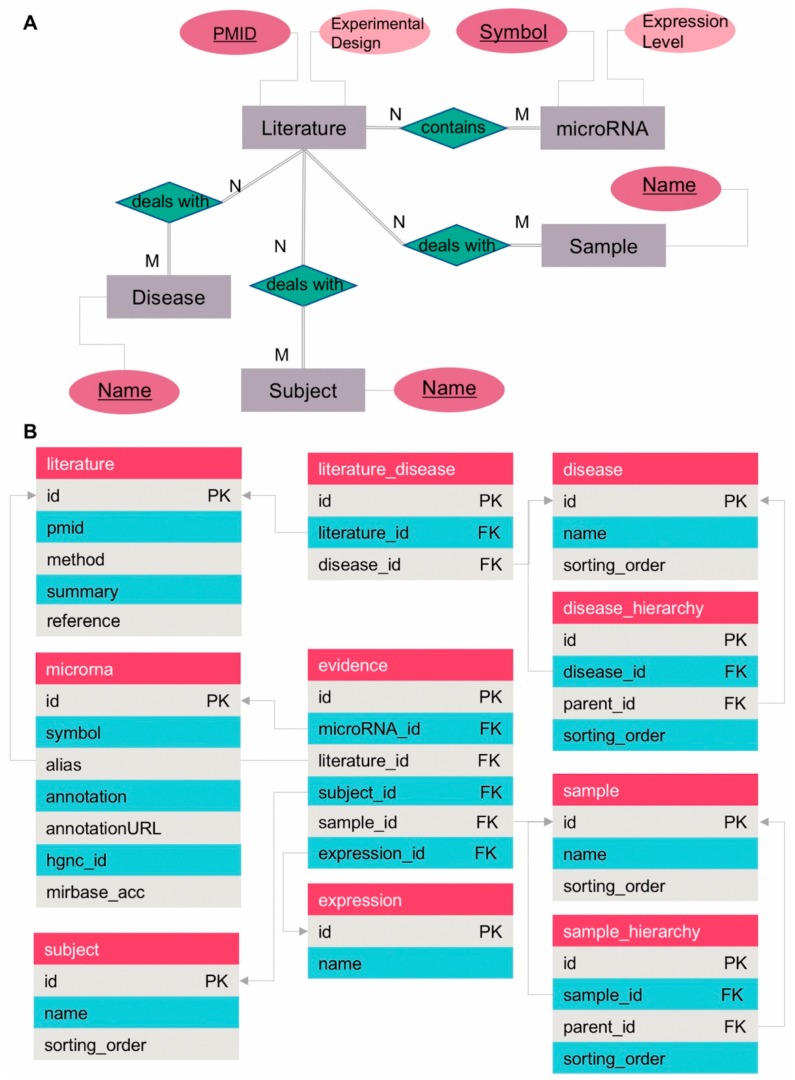
Our database model diagrams. (**A**) An entity relationship (ER) model diagram of miR2Diabetes, using the notation of Chen [23]. Gray squares represent entities and dark green diamond shapes are relationships. An ellipse shape is an attribute, divided such that a dark pink ellipse shape is a key attribute, and a pink ellipse shape is an important attribute to handle. (**B**) A table-like model diagram of miR2Diabetes. A dark pink square represents a table name. Gray and cyan squares are fields of a table. PK means a primary key of its table and FK means a foreign key to another table (indicated by arrows). Detailed definitions of our table-like model are downloadable from the miR2Diabetes website [22].

We implemented our database in Oracle™ MySQL 5.5. Figure 2 shows an entity-relationship model (ERD), and a table-like model. Using “Chen ERD notation” [23] to represent our ERD, we first identified five entities (grey boxes in Figure 2A) with four relationships (green diamonds in Figure 2A). Based on our ERD (Figure 2A), we constructed a table-like model (Figure 2B). The entity ‘Literature’ (Figure 2A) had many-to-many relationships with the four other entities. We split the ‘Disease’ entity, in Figure 2A, into two tables, ‘disease’ and ‘literature_disease’ in Figure 2B. The ‘literature_disease’ table in Figure 2B is a look-up table for many-to-many relationships for all the entities of Figure 2A. Similarly, we created an ‘evidence’ table to connect between ‘literature,’ ‘microrna’, ‘subject’, ‘sample’, and ‘expression’ tables in Figure 2B. In addition, we added two tables, ‘disease_hierarchy’ and ‘sample_hierarchy’, creating a recursive data structure. The two tables aimed to represent the hierarchical taxonomy structure of the disease ontology [20] and the BRaunschweig ENzyme DAtabase (BRENDA) Tissue and Enzyme Source Ontology [19]. It is noted that, for being self-evident, we did not present field types in the table-like model (Figure 2B). The data definition language script is available at the miR2Diabetes website [22]. 

### 3.3. Database Access and Case Study of miR2Diabetes

**Figure 3:**
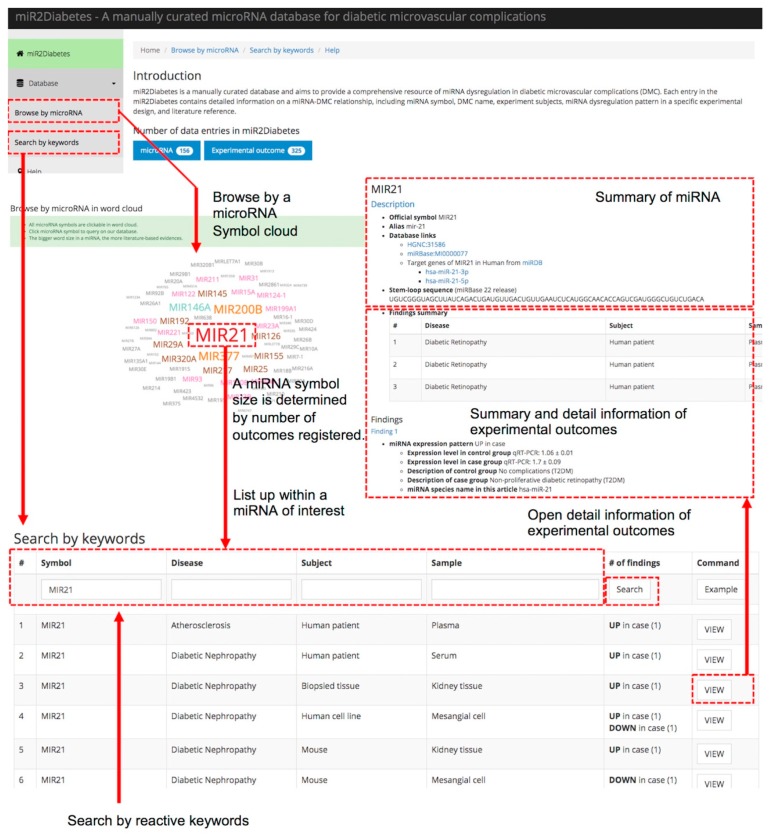
Schematic workflow of the miR2Diabetes database. As shown, the user may browse by microRNA names or by keywords. Selection of specific microRNAs (with word sizes based on publication occurrences) provides a summary of specific study outcomes and other detailed information. Filters provided include miRNA, disease, subject, type of tissue sample, and up- vs. down-regulation.

We designed our miR2Diabetes database to provide a modern and comprehensive user experience for browsing data by miRNA symbols and/or searching by keywords (Figure 3). Moreover, the user can access data by retrieving pages through a navigation menu at the left side of our web page, or via a breadcrumb menu at the top of a content page. For instance, the ‘Browse by microRNA’ menu item leads the user to a miRNA cloud, with the size of symbol indicating the number of experimental outcomes associated with that miRNA. Likewise, clicking a miRNA gene symbol of interest (e.g., MIR21) opens a list of relative observations extracted from the literature. It is also possible to specify certain entries of interest by typing keywords into the input boxes on the list page. 

In extension to the above, user typing in the auto-fill input boxes help users by suggesting data entries containing the user’s entered keyword, as does the ‘Search by keywords’ menu item (via keyword input boxes). For example, a user typing ‘diabetic’ in the disease input box (Figure 3) will retrieve disease entries that incorporate that keyword. According to specified miRNA symbols, or keywords, our database returns a list of matched entries, which may be further filtered by additional keywords. For example, after clicking ‘MIR21’ from the ‘Browse by microRNA’ page, the results can be further narrowed down by selecting ‘Diabetic Nephropathy’ in the ‘Disease’ input box, and ‘Human patient’ in the ‘Subject’ input box.

Additional features are provided for different types of searches. One such feature is that from the search results page, clicking the ‘VIEW’ button to the right of the list leads to more detailed information about the experimental outcome. This page provides two main types of information to users. At the top of the content page, there is a summary of microRNAs, including official symbols, a list of aliases, a list of links to other databases, and an annotation. At the bottom of the miRNA section is a “summary of outcomes” table for user convenience. The rest of the content page contains detailed information on experimental outcomes, including miRNA dysregulation patterns, diseases, experimental subjects, and other entities, including published references. In addition, it is possible to jump to a specific miRNA dysregulation observation section by clicking the ‘Go’ internal link button, and to return by clicking by the ‘Back to top’ internal anchor at the end of the observation section. At the top of the content view page, clicking the ‘Go back’ anchor returns to the results list page.

## 4. Discussion and Conclusions

DM is a significant societal burden that strongly evokes a myriad of complications (DMCs). In 2017, worldwide, DM afflicted over 420 million (8.8% lifetime risk), at a direct healthcare cost of $673 billion USD, and five million deaths [24]. Moreover, those statistics are expected to increase to an incidence of 642 million (10.4% lifetime risk), at a cost of $802 billion USD, by 2040 [2]. Like most human diseases, DM and DMCs strongly associate with dysregulation of miRNA expression, but unlike cancer databases [25,26], there are no major DM/DMC genomic/transcriptomic research repositories. To date, there exist 65 observations of miRNAs in urine, and 42 observations in plasma and serum, with all three representing 49.53% of all published miRNA studies. This fact likely reflects increased interest in investigating miRNAs as reliable DM and DMC biomarkers, even while there exists only one disease-related miRNA database [12] of 15 DM/DMC-associated miRNA entries, and these may be outdated (2009). Thus, it is important to build a DM- and DMC-domain-specific database, to provide highly biocurated contents, with a focus on certain systemic diseases, even while these may be limited in numbers of data entries. 

Considering DM and DMC as emerging health problems, either preventing progression of DM to DMCs or detecting DM or DMCs, at an early stage, is paramount. To that end, diverse biomarker development has been attempted, but current biomarkers (e.g., glycated hemoglobin, blood glucose) have limited success. As miRNAs have been regarded as ideal disease biomarker candidates (based on their stability and consistency) [27], our database enables investigators to inspect feasibility of their own miRNA biomarker candidates in DM and DMCs, before in-vivo and in-vitro validations. Our study did not aim at in-depth methodology of the miRNA expression patterns. Instead, our study focused on accurate curation that is critical for biological databases, in terms of integrity of database content [28]. In that capacity, our miR2Diabetes repository provides accurately curated information of experimentally DM- and DMC-relating miRNAs. In summary, we believe that our newly developed miR2Diabetes database represents a valuable compendium for identifying DM- and DMC-related miRNA dysregulation patterns, in the field of translational endocrinology researchers, providing appreciable insight into the pathogenic mechanisms of these devastating diseases. 

## Database Availability

miR2Diabetes [22].
